# When Should Spinal Surgery Be Considered for Spinal Metastasis from Non-Small Cell Lung Cancer?—Propensity Score Matched Study between Surgery with Radiotherapy and Radiotherapy Alone

**DOI:** 10.3390/jcm12144683

**Published:** 2023-07-14

**Authors:** Hyung-Youl Park, Kee-Yong Ha, Sang-Il Kim, Yeon-Sil Kim, Yongwon Joh, Young-Hoon Kim

**Affiliations:** 1 Department of Orthopaedic Surgery, Eunpyeong St. Mary’s Hospital, College of Medicine, The Catholic University of Korea, Seoul 03312, Republic of Korea; matrixbest@naver.com (H.-Y.P.);; 2 Department of Orthopedic Surgery, Kyung Hee University Hospital at Gangdong, Seoul 05278, Republic of Korea; 3 Department of Orthopedic Surgery, Seoul St. Mary’s Hospital, College of Medicine, The Catholic University of Korea, Seoul 06591, Republic of Korea; 4 Department of Radiation Oncology, Seoul St. Mary’s Hospital, College of Medicine, The Catholic University of Korea, Seoul 06591, Republic of Korea

**Keywords:** lung neoplasms, surgery, radiotherapy, molecular targeted therapy, neurologic manifestations, survival rate

## Abstract

(1) Background: Although metastatic spine disease is increasing, the debate on therapeutic modality remains due to the heterogeneity of tumors and patients. This study aims to evaluate the efficacies of surgery and risk factors for patients’ survival from potentially unstable spinal metastasis of non-small cell lung cancer; (2) Methods: Twenty-two patients undergoing surgery and radiotherapy (group I) were compared with 22 patients undergoing radiotherapy alone (group II) using propensity score matching in a 1-to-1 format. Clinical outcomes included the performance status and ambulatory status. In addition, independent risk factors for patients’ survival were evaluated, including the molecular targeted therapy for mutations; (3) Results: deterioration in neurologic status was only observed in group II compared to group I (22.7% vs. 0%, *p*-value = 0.018). In addition, five patients in the surgery group showed improved Frankel grades. Regarding the patients’ survival, a smoking history of more than ten pack-years (hazard ratio (HR) = 12.18), worse performance status (HR = 6.86), and absence of mutations (HR = 3.39) were the independent risk factors; (4) Conclusions: Spinal surgery with radiotherapy could have advantages for improving the neurologic status including ambulation for potentially unstable spine due to metastasis. Thus, surgery should be considered for patients with a longer life expectancy resulting from better performance status and use of the targeted therapy.

## 1. Introduction

The incidence of metastatic spine disease (MSD) is increasing due to longer life expectancy and improvements in medical treatment for cancer patients [1,2,3]. Spinal metastasis affects up to 70% of cancer patients, but symptoms appear in 10–20% of patients, causing pain, neurologic deficits, and poor quality of life [2]. Thus, palliative surgery for MSD provides neural decompression for cord compression and mechanical stability for an unstable spine, resulting in increased quality of life, such as pain control and self-ambulation [4,5].

In a breakthrough study in MSD, Patchell et al. demonstrated the efficacy of surgery with postoperative radiotherapy superior to radiotherapy alone for patients with MSD [6]. In addition, many studies have reported that surgery with radiotherapy produces better clinical outcomes, including ambulation and survival, than radiotherapy alone [7,8]. However, the role of surgery on MSD is still debated because the primary origin sites of tumors and patients’ conditions are different according to the studies [9].

It is unavoidable that the overall survival rate of MSD patients is still poor. However, molecular targeted therapies, referring to drugs that interfere with specific molecules to block the growth and spread of cancer cells, have demonstrated remarkable clinical success in cancer treatment, including lung, breast, colorectal, and ovarian tumors [10]. Recent studies also reported that the median survival of MSD patients treated with molecular targeted therapy was better [11,12].

The purpose of this study was to evaluate the clinical efficacy of spinal surgery with radiotherapy compared to radiotherapy alone using propensity score-matched analysis, limited to spine metastasis of non-small cell lung (NSCLC) cancer, to minimize differences in patient’s condition and tumor heterogeneity. In addition, the risk factors for patients’ survival were assessed, including the molecular targeted therapy for mutations. Therefore, this study aims to suggest an appropriate indication of surgical treatment in patients with spinal metastasis of NSCLC.

## 2. Materials and Methods

### 2.1. Propensity Score-Matched Cohort

A clinical database was retrospectively reviewed from 2011 to 2017 at a single tertiary institution. The patients treated with surgery combined with postoperative radiotherapy (surgery group) for the spine metastasis of lung cancer were included. In addition, patients with a tissue-proven diagnosis of adenocarcinoma and MRI evidence were eligible for the study [13]. Thirty-seven patients underwent spinal surgery with radiotherapy for lung cancer metastasis in our institution. A total of 231 patients were treated with radiotherapy alone for spinal metastasis of NSCLC, especially for adenocarcinoma. We included the patients treated with surgery or radiotherapy for thoracic spine metastasis to evaluate their ambulatory status. The patients with concomitant skeletal metastasis treated with separate radiotherapy were excluded.

**Figure 1:**
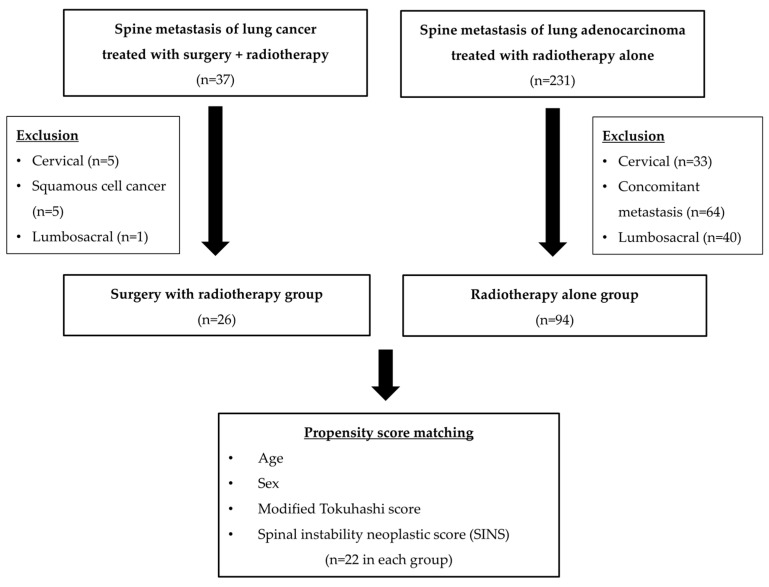
Flow chart of patient inclusion through propensity score matching.

Twenty-six patients in the surgery group were matched with 94 patients who underwent only radiotherapy (only RT group) for spinal metastasis of lung adenocarcinoma. A multivariate logistic regression test was used to calculate propensity score according to age, sex, modified Tokuhashi score, and spinal instability neoplastic score (SINS) [14,15]. Patients in the surgery group were matched 1:1 to patients in the only RT group with a caliper of 0.2 times the propensity score’s standard deviation (SD). Four people in the surgery group were not matched among the patients undergoing only RT. Twenty-two patients in each group were included in this study according to inclusion and exclusion criteria (Figure 1). The institutional review board approved the study protocol (KC18RESI0605), ensuring compliance with the ethical guidelines of the 1975 Helsinki Declaration. In addition, because of the retrospective study design, the institutional review board waived the need to obtain informed patient consent.

### 2.2. Procedures of Surgery and Radiotherapy

The purpose of the surgery was to provide decompression of the spinal cord and stabilization using bone grafting and fixation devices in a palliative setting [2]. The surgical procedures were tailored for each patient depending on the location of the tumors and the patient’s condition [16]. In general, anterior corpectomy or posterior vertebral column resection (PVCR) with interbody bone grafting was performed for anteriorly located tumors with vertebral body collapse and spinal instability [17]. Decompression with additional instrumentation was carried out for patients with epidural spinal cord compression but normal spinal alignment without vertebral body collapse and instability [18].

Conventional RT or intensity-modulated radiation therapy (IMRT) other than stereotactic body radiation therapy (SBRT) was conducted similarly for both the surgery group and the only RT group. The total dose was 30–45 Gy given 10–15 fractions depending on the patient’s functional status and extent of spinal metastasis. Treatments were delivered to a port encompassing one vertebral body above and below the visible lesion after performing the simulation CT [19].

### 2.3. Outcome Measurements

Regarding the neurologic status, orthopedic surgeons and physiatrists evaluated the ability to ambulate. A patient was deemed ambulatory if patients could take at least two steps with each foot unassisted (a total of four steps), even if a cane or walker were needed [6]. The Frankel grade was also assessed to range from A to E according to the degree of neurologic deficits (A = complete, B, C, D = incomplete, E = normal) [20]. Performance status was evaluated using Eastern Cooperative Oncology Group (ECOG)—performance status (PS) ranging from 0 to 4 (a higher score on ECOG-PS indicates poorer performance status) [21]. Neurological and performance status assessments were performed before the treatment and at the last follow-up. Patients had regular follow-up assessments every 4-6 weeks until the end of the study or death.

Clinical outcomes, including ambulation, Frankel grade, and ECOG-PS, were compared between the surgery group and the only RT group. Moreover, univariate and multivariate analyses were carried out to identify the risk factors for the patient’s survival, including mutations of epidermal growth factor receptor (EGFR) or anaplastic lymphoma kinase (ALK) [22,23].

### 2.4. Statistical Analyses

Perioperative continuous variables, presented as the means and SDs, were compared using the Student’s *t*-test or the Wilcoxon rank sum test according to the Kolmogorov-Smirnov test. Categorical variables were compared using Pearson’s chi-square test or Fisher’s exact test, depending on the distribution of the samples. Variable with *p*-value < 0.1 on univariate analysis was included in multivariate analysis using logistic regression test. The cumulative survival rate was analyzed using Kaplan–Meier survivorship analysis with a log-rank test. Statistical analysis was conducted using the SPSS software (IBM SPSS Statistics, Version 24.0, Armonk, NY, USA: IBM Corp.) with a significant level of 0.05.

## 3. Results

### 3.1. Patients Undergoing Spinal Surgery for Metastatic NSCLC

Nine patients underwent anterior corpectomy or PVCR with interbody cage insertion and posterior fusion with or without decompression. Thirteen patients underwent the posterior laminectomy with instrumentation. Pre-operative embolization to reduce intraoperative bleeding was carried out in eight patients. The mean operation time was 175.5 ± 45.3 min. Average intra-operative bleeding was 679.6 ± 435.8 mL, and transfusion was 565.7 ± 433.7 mL. Regarding complications, one patient experienced post-operative wound infection, treated with intravenous antibiotics without reoperation. However, radiotherapy was delayed due to a wound problem.

### 3.2. The Comparison between the Surgery with Radiotherapy and Radiotherapy Alone

Patient demographics and clinical outcomes in both groups are shown in Table 1. Matched parameters such as age, sex, modified Tokuhashi score, and SINS were similar between the two groups. Average SINS were 9.6 in the surgery group and 10.1 in the only RT group. Both values between 7 to 12 points mean a potentially unstable spine due to metastatic lung cancer. Bone mineral density (BMD) was also similar in both groups (−2.6 ± 0.1 vs. −2.7 ± 0.1, *p* = 0.848). Regarding the characteristics of lung cancer, EGFR or ALK mutations and smoking history were similar between the two groups (45.5% vs. 31.8%, *p* = 0.353 for mutations; 13.0 ± 20.0 vs. 13.3 ± 19.9 pack-year, *p* = 0.958 for smoking).

Before the treatment, the number of patients able to ambulate and good performance status (ECOG-PS 0, 1, 2) was significantly greater in the only RT group compared to the surgery group (95.5% vs. 72.7%, *p* = 0.039 for ambulation; 95.5% vs. 72.7%, *p* = 0.039 for good ECOG-PS, respectively). However, worse Frankel grade (A, B, C) was significantly more common in the surgery group than in the only RT group (27.3% vs. 4.5%, *p* = 0.039). At the last follow up, neurologic parameters and ECOG-PS were similar in both groups (all *p*-values > 0.05). Both groups had similar the period of follow up (12.3 ± 11.8 vs. 11.3 ± 14.7 months, *p* = 0.813) 

**Figure 2:**
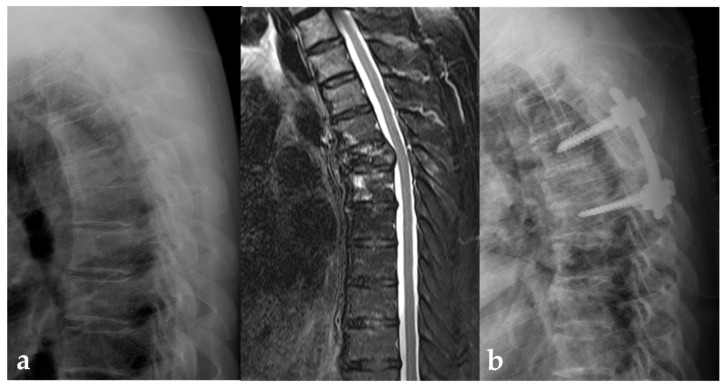
(**a**) A 54-year old female patient treated for NSCLC unable to ambulate with Frankel grade C. Imaging studies reveal the D5 pathologic fracture and epidural spinal cord compression. (**b**) After the patient underwent posterior instrumentation and neural decompression followed by radiotherapy, she could ambulate and survived for two years with molecular targeted therapy for the EGFR mutation.

**Figure 3:**
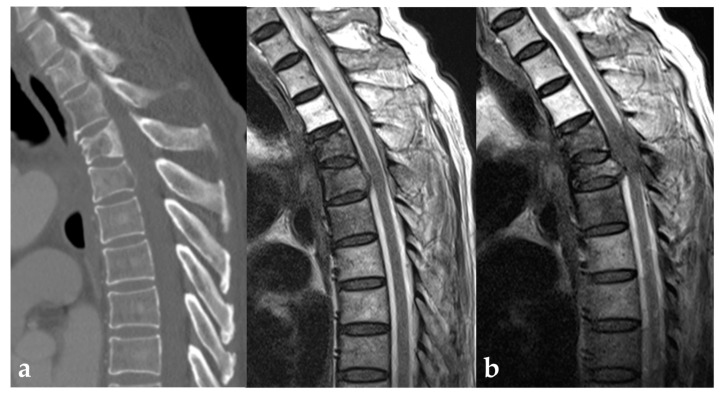
(**a**) A 63-year old male patient confirmed with NSCLC presented with Frankel grade E. Imaging studies reveal the D4 pathologic fracture and mild cord compression. (**b**) After the radiotherapy, Frankel grade deteriorated due to progressive pathologic fracture and severe epidural spinal cord compression, and the patient expired after three months.

Neurologic status and changes at the initial and last follow up are presented in Table 2. In the surgery group, improvement of Frankel grade was observed in 5 patients, and four patients unable to ambulate were able to walk at the last follow up (Figure 2). Instead, five patients in the only RT group presented with deteriorations, while no deterioration in the surgery group (22.7% vs. 0%, *p* = 0.018) (Figure 3).

However, cumulative survival rates were similar between the surgery group and the only RT group (*p* = 0.790 on the log-rank test). Median survival time in patients undergoing spinal surgery with RT was 19.0 months (95% confidence interval [CI], 0–41.3) and 8.0 months in patients undergoing radiotherapy alone (95% CI: 0–22.9).

### 3.3. Analysis of Risk Factors for the Patient’s Survival

Univariate analysis for patient survival is presented in Table 3. Patients with a smoking history of more than ten pack-years had shorter median survival than those with less than ten pack-years (5.0 vs. 36.0 months, *p* = 0.005). Worse ECOG-PS (3–4) and ambulation at the last follow up was also the risk factor for survival (3.0 vs. 21.0 months, *p* = 0.000 for ECOG-PS; 2.0 vs. 19.0 months, *p* = 0.002 for ambulation). Patients treated with targeted molecular therapy for mutation of EGFR or ALK had significantly longer survival than patients undergoing conventional chemotherapy without mutation (21.0 vs. 5.0 months, *p* = 0.042). Other parameters such as sex, age, ECOG-PS, and ambulation status before the treatment were not significant risk factors for survival.

**Figure 4:**
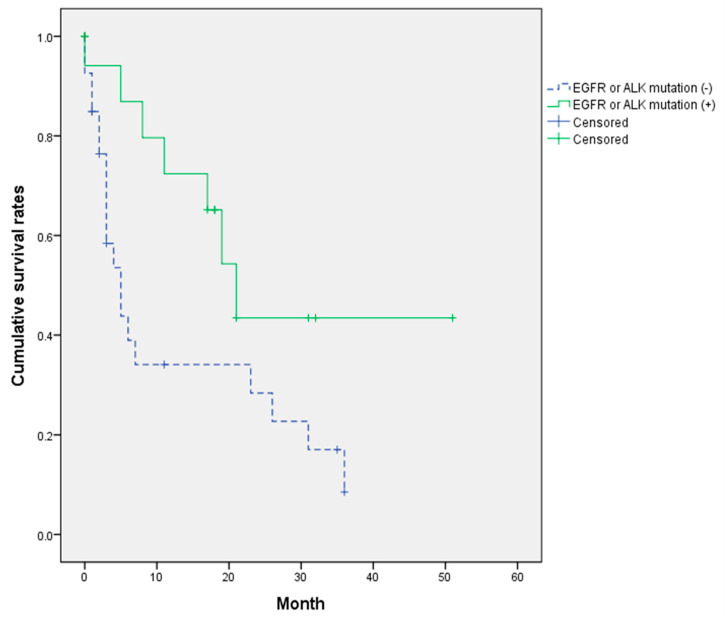
Cumulative survival rates according to the targeted molecular therapy for EGFR or ALK mutations.

Multivariate analysis using logistic regression is presented in Table 4. A more than ten-pack-year smoking history was the independent risk factor for patient survival (hazard ratio (HR) = 12.18, 95% CI: 2.13–69.7). In addition, worse ECOG-PS (3–4) at the last follow up was also the risk factor for survival (HR = 6.86, 95% CI: 0.13–102.5). Patients undergoing conventional chemotherapy without EGFR or ALK mutation had significantly shorter survival than those treated with molecular targeted therapy (HR = 3.39, 95% CI: 0.95–12.1). Cumulative survival rates were also significantly different according to the EGFR or ALK mutations (*p* = 0.042 on the log-rank test) (Figure 4). Median survival time in patients with mutations was 21 ± 2.8 months (95% CI: 15.5–26.5) and 5 ± 1.4 months in patients without mutations (95% CI: 2.2–7.8).

## 4. Discussion

Traditional decompressive surgery without stabilization for metastatic spine disease (MSD) resulted in worse outcomes, so conventional radiotherapy was preferred [24]. However, surgery for MSD has been increasing because of the development of surgical techniques and the increasing incidence of MSD [18,25,26]. Moreover, recent studies have shown that surgery with radiotherapy for MSD results in better outcomes, including quality of life, than radiotherapy alone [6,7]. Surgery’s role in MSD is appropriately discussed in specific situations because tumor origin sites and patients’ conditions differ [16]. Therefore, this study was designed to evaluate the role of surgery compared to radiotherapy alone, confined to patients with thoracic spine metastasis of NSCLC and similar conditions by propensity score matching. Propensity scores were calculated by age, sex, modified Tokuhashi score, and SINS and reflected patients’ general conditions and spinal instability.

Patient demographics, such as BMD and mutations of EGFR or ALK, and matched parameters were similar in both groups. In matched cohort, the patients with better ECOG-PS (0, 1, 2) and Frankel grade (D, E) before treatment were significantly more common in the only RT group (95.5% vs. 72.7%). In contrast, the number of patients unable to ambulate was significantly greater in the surgery group (27.3% vs. 4.5%). However, those parameters at the last follow up were similar in both groups (*p* > 0.05 for all parameters). In the surgery group, improvements in ambulation and Frankel grade were observed (four patients for ambulation and five for Frankel grade). The surgery with radiotherapy could improve the neurologic statuses by direct neural decompression. Although a direct comparison of neurologic recovery between the surgery group and RT alone group was not possible due to different neurologic statuses before the treatment, a previous study reported that relief of Frankel grade in the surgery group was superior to that of the radiotherapy group (*p* = 0.025) in treating spinal metastasis of lung cancer [27]. In the pathologic vertebral fracture in multiple myeloma, we also reported that spinal surgery might provide better clinical outcomes than RT alone in maintaining independent ambulation, neurological status, and pain control despite similar median survival and complications [28].

More importantly, the deterioration of Frankel grade was found only in five patients treated with radiotherapy alone compared to that of no patient in the surgery group. All five patients presented de novo or progressive spine pathologic fractures after the radiotherapy. Lee et al. reported that vertebral compression fracture was developed as a delayed consequence of spinal RT up to 14.8%. Risk factors were spinal metastasis with SINS > seven and baseline compression fracture [29,30]. Our study also revealed that surgery provided better clinical outcomes through pathological fracture prevention in the potentially unstable spine, where SINS was 9.6 and 10.1 points in each group. This result was consistent with our previous report regarding multiple myeloma. In patients with unstable pathologic vertebral fractures due to multiple myeloma, spinal surgery with adjuvant RT compared with RT alone provided better clinical outcomes in terms of maintaining independent ambulation and neurologic status despite similar median survival and complications [28]. In this regard, surgery for the MSD of lung cancer should be considered for cases of potentially unstable spine and neurological deficits that can be resolved by direct decompression.

Despite these advantages of surgery regarding neurologic recovery and preservation in our study, the survivals were similar between the surgery group and the only RT group (19.0 vs. 8.0 months, *p* = 0.790). Zhang et al. demonstrated that surgery with postoperative radiotherapy did not significantly prolong survival compared to standalone radiotherapy for patients with spinal metastasis of lung cancer [27]. Previous studies have reported that the most important survival factors for metastatic lung cancer are systemic chemotherapy rather than local therapy such as surgery or radiotherapy [31,32]. However, some studies are contrary to these results. Two meta-analyses have reported that direct decompressive surgical resection followed by radiotherapy might provide better survival rates than radiotherapy alone in the treatment of metastatic epidural spinal cord compression [7,8]. Therefore, further trials are needed to conclude the efficacy of the surgery on survivals of MSD.

In multivariate analysis, more than a ten-pack-year smoking history (HR = 12.18), worse ECOG-PS (3–4) at the last follow up (HR = 6.86), and conventional chemotherapy without EGFR or ALK mutation (HR = 3.39) were the independent risk factors for patient survival. Except for smoking history, better performance status at the last follow up and targeted therapy for EGFR or ALK mutation significantly improved survival rates for metastasis of lung cancer. Good performance status (0, 1, 2) is essential to maintain systemic chemotherapy, including targeted therapy to prolong survival for lung cancer treatment [33]. In a meta-analysis, Tan et al. also reported that maintenance treatments using EGFR inhibitors showed clinically meaningful survival benefits in suitable performance status patients, including 12 trials for 3850 patients [34].

In this regard, cumulative survival rates were also significantly different. The median survival time in the molecular targeted therapy group was significantly longer (21 ± 2.8 vs. 5 ± 1.4 months, *p* = 0.042 on log-rank test) in our study. This finding is interesting because the mean Tokuhashi in included cohorts was 5.2 points, meaning that expected life expectancy was less than six months. It is consistent with a previous report that positive EGFR mutation provided longer postoperative survival for NSCLC patients undergoing palliative surgical treatment for spinal metastases (HR = 2.10, *p* = 0.002) [35]. Furthermore, Batista et al. demonstrated that the median survival of patients treated with EGFR inhibitors was improved up to 18 months. In comparison, the overall median survival of patients with spine metastasis of lung cancer was poor, ranging from 3.6 to 9 months [12]. Better performance status for patients treated with molecular targeted therapy for mutations might result in significantly longer survival than those treated with conventional chemotherapy, causing poor performance status in this study. Therefore, biological treatment for NSCLC should be considered in the decision-making process for spinal metastasis.

This study has some limitations. First, propensity score matching considering the patients’ general conditions and spinal instability other than neurologic status was done to consider the challenging clinical situations. The neurologic statuses at the initial presentation, such as ambulation and Frankel grade, were different between the two groups. Thus, a direct comparison of the neurologic recovery between the two groups was not possible, and the possibility of selection bias could be in this study. Second, many factors not included in the analysis might have affected outcome parameters. We excluded functional outcomes such as pain, disability, and patients with concurrent skeletal metastasis treated with separate radiotherapies. Third, SBRT has been rapidly developing and applied to treat MSD [36]. However, the impact of the SBRT on MSD was not evaluated in this study. Finally, recent studies have reported that spinal surgery could improve the neurologic status, quality of life, and survival in the treatment of MSD [7,8]. Our study also revealed that ECOG-PS at the last follow up was the significant factor in survival. The small number of patients and selection bias in this study did not conclude the efficacy of the surgery on the survivals. Therefore, further trials are needed to assess the efficacy of the surgery with a large number of cohorts for the MSD of lung cancer, including a comparison between surgery and SBRT.

Despite these limitations, this study has the following strengths. First, the results were from the patients with potentially unstable spines (SINS between 7 and 12 points) who are clinically challenging in deciding the treatment option. Radiotherapy is considered for stable spines with a SINS lower than six points, and surgical treatment is considered for unstable spines with a SINS higher than 13 points [15]. Our study revealed that surgery with radiotherapy should be considered for cases of a potentially unstable spine with the possibility of pathologic fractures and neurological deficits that can be resolved by direct decompression. Second, patients treated with targeted therapy for EGFR or ALK mutation had longer survivals than life expectancy (<6 months) due to the modified Tokuhashi score. Therefore, biological treatment for NSCLC should be considered in the decision-making process for spinal metastasis.

## 5. Conclusions

This study revealed that spinal surgery combined with radiotherapy maintained the neurologic status compared to the radiotherapy alone group in the potentially unstable spine due to metastasis of NSCLC. In addition, the surgery improved ambulation and Frankel grade in patients with neurologic deficits. In contrast, better performance status and molecular targeted therapy significantly improved the survival rate. Therefore, in metastatic NSCLC, patients with neurologic deterioration and potentially unstable spine should be considered for surgery combined with radiotherapy, especially in patients with mutations of EGFR or ALK expected to have a longer life expectancy despite a lower Tokuhashi score.

## Figures and Tables

**Table 1:** Patient demographics and clinical outcomes according to the treatment.

	Surgery + RT	RT Alone	*p*-Value
Number of patients	22	22	
Age	64.1 ± 11.4	63.9 ± 10.0	0.944
Sex (Male)	13 (59.1%)	15 (68.2%)	0.531
Tokuhashi score	5.4 ± 2.2	5.0 ± 1.8	0.549
SINS	9.6 ± 2.2	10.1 ± 2.7	0.541
BMD	−2.6 ± 0.1	−2.7 ± 0.1	0.848
EGFR or ALK mutation	10 (45.5%)	7 (31.8%)	0.353
Smoking (pack-year)	13.0 ± 20.0	13.3 ± 19.9	0.958
Initial			
Ambulation	16 (72.7%)	21 (95.5%)	0.039
Frankel grade (A, B, C)	6 (27.3%)	1 (4.5%)	0.039
ECOG-PS (0, 1, 2)	16 (72.7%)	21 (95.5%)	0.039
Last follow up			
Ambulation	18 (81.8%)	17 (77.3%)	1.000
Frankel grade (A, B, C)	2 (9.1%)	2 (9.1%)	1.000
ECOG-PS (0, 1, 2)	15 (68.2%)	16 (72.7%)	0.741
Follow-up period (months)	12.3 ± 11.8 (0–35)	11.3 ± 14.7 (0–51)	0.813

Note: RT—radiotherapy; SINS—spinal instability neoplastic score; BMD—bone mineral density; EGRF—epidermal growth factor receptor; ALK—anaplastic lymphoma kinase; ECOG—Eastern Cooperative Oncology Group; PS—performance status.

**Table 2:** Changes in neurologic status according to the treatment.

Parameters		Group I (Surgery + RT, n = 22)					Group II (RT Alone, n = 22)					*p*-Value
		Frankel Grade (Last F/U)										
		A	B	C	D	E	A	B	C	D	E	
Frankel grade (initial)	A											
	B		2									
	C				2	2	1					
	D				2	1			1	1		
	E					13				3	16	
Deterioration												
Ambulation		3 (13.6%)					4 (18.2%)					1.000
Frankel grade		0 (0%)					5 (22.7%)					0.018
ECOG-PS		7 (31.8%)					6 (27.3%)					0.741

Note: RT—radiotherapy; F/U—follow up; ECOG—Eastern Cooperative Oncology Group; PS—performance status.

**Table 3:** Univariate analysis for the risk factors of the patient’s survival.

	Number of Patients	Median Survival (95% CI)	*p*-Value (log-Rank)
Treatment			
Surgery + RT	22	19.0 (0.0–41.3)	0.790
RT alone	22	8.0 (0.0–22.9)	
Sex			
Male	28	7.0 (1.2–12.7)	0.280
Female	16	19.0 (1.1–36.9)	
Age			
≥60 years	22	8.0 (0.0–22.9)	0.790
<60 years	22	19.0 (0.0–41.3)	
Smoking history			
≥10 pack-year	17	5.0 (3.1–6.8)	0.005
<10 pack-year	27	36.0 (11.6–60.4)	
ECOG-PS (initial)			
0–2	37	11.0 (0.0–27.9)	0.999
3–4	7	36.0 (-)	
ECOG-PS (last F/U)			
0–2	31	21.0 (13.6–28.4)	0.000
3–4	13	3.0 (1.6–4.4)	
Ambulatory status (initial)			
Ambulatory	37	11.0 (0.0–27.9)	0.999
Non-ambulatory	7	36.0 (-)	
Ambulatory status (last F/U)			
Ambulatory	35	19.0 (4.9–33.1)	0.002
Non-ambulatory	9	2.0 (0.0–4.9)	
Frankel grade (initial)			
D, E	37	36.0 (–)	0.999
A, B, C	7	11.0 (0.0–27.9)	
Frankel grade (last F/U)			
D, E	40	17.0 (2.3–31.7)	0.237
A, B, C	4	3.0 (0.5–5.5)	
EGFR or ALK mutation			
Yes	17	21.0 (15.5–26.5)	0.042
No	27	5.0 (2.1–7.8)	

Note: RT—radiotherapy; ECOG—Eastern Cooperative Oncology Group; PS—performance status; F/U–follow up; EGRF—epidermal growth factor receptor; ALK—anaplastic lymphoma kinase.

**Table 4:** Multivariate analysis for the risk factors of the patient’s survival.

	Odds Ratio	95% CI	*p*-Value
Smoking history			
≥10 pack-year	12.18	2.13–69.7	0.005
<10 pack-year	-		
ECOG-PS (last F/U)			
0–2	-		0.037
3–4	6.86	1.12–42.12	
Ambulatory status (last F/U)			
Ambulatory	-		0.456
Non-ambulatory	3.58	0.13–102.5	
EGFR or ALK mutation			
Yes	-		0.05
No	3.39	0.95–12.1	

Note: ECOG—Eastern Cooperative Oncology Group; PS—performance status; F/U—follow up; EGRF—epidermal growth factor receptor; ALK—anaplastic lymphoma kinase.

## Data Availability

Original data will be made available upon reasonable request.
